# Test-Retest reliability and preliminary reliable change estimates for Sway Balance tests administered remotely in community-dwelling adults

**DOI:** 10.3389/fdgth.2022.999250

**Published:** 2022-11-02

**Authors:** Jaclyn B. Caccese, Elizabeth Teel, Ryan Van Patten, Mélissa A. Muzeau, Grant L. Iverson, Heidi A. VanRavenhorst-Bell

**Affiliations:** ^1^ School of Health & Rehabilitation Sciences and Chronic Brain Injury Program, The Ohio State University College of Medicine, Columbus, OH, United States; ^2^ School of Physical and Occupational Therapy, McGill University, Montreal, QC, Canada; ^3^ Providence Veterans Administration Medical Center, Providen, RI, United States; ^4^ Department of Psychiatry and Human Behavior, Alpert Medical School of Brown University, Providence, RI, United States; ^5^ Sporttesting, Grenoble, France; ^6^ Human Performance Laboratory, Wichita State University, Wichita, KS, United States; ^7^ Department of Physical Medicine and Rehabilitation, Harvard Medical School, Boston, MA, United States; ^8^ Department of Physical Medicine and Rehabilitation, Spaulding Rehabilitation Hospital, Charlestown, MA, United States; ^9^ Sports Concussion Program, MassGeneral Hospital for Children, Boston, MA, United States; ^10^ Department of Physical Medicine and Rehabilitation, Schoen Adams Research Institute as Spaulding Rehabilitation, Charlestown, MA, United States; ^11^ Home Base, A Red Sox Foundation and Massachusetts General Hospital Program, Charlestown, MA, United States; ^12^ Department of Human Performance Studies, Wichita State University, Wichita, KS, United States

**Keywords:** postural stability, balance error scoring system (BESS), balance, concussion, psychometrics (MeSH)

## Abstract

**Objective:**

Impaired balance and postural stability can occur with advanced age, following traumatic brain injury, in association with neurological disorders and diseases, and as the result of acute or chronic orthopedic problems. The remote assessment of balance and postural stability could be of value in clinical practice and research. We examined the test-retest reliability and reliable change estimates for Sway Balance Mobile Application tests (Sway Medical, Tulsa OK, USA) administered remotely from the participant's home.

**Method:**

Primarily young, healthy community-dwelling adults completed Sway Balance Mobile Application tests remotely on their personal mobile devices once per week for three consecutive weeks while being supervised with a video-based virtual connection. Sway Balance tests include five stances (i.e., feet together, tandem right foot forward, tandem left foot forward, single leg right foot, single leg left foot), which are averaged to compute a Sway Balance composite score from 0 to 100, with higher scores indicating better postural stability. We examined test-retest reliability (measured with intraclass correlation coefficients, ICCs) and preliminary reliable change estimates for 70%, 80%, and 90% confidence intervals.

**Results:**

Participants included 55 healthy adults (ages = 26.7 ± 9.9 years, interquartile range = 20–30, range = 18–58; 38 [69%] women). Test-retest reliability for the Sway Balance composite score across three weeks was.88. Test-retest reliability for individual stances ranged from 62 to 83 (all ps < 0.001). At the 80% confidence interval, preliminary reliable changes estimates were 9 points for the Sway Balance composite score.

**Conclusions:**

For a remote administration, test–retest reliability was moderate-to-good for all Sway Balance stances, as well as for the Sway Balance composite score. Reliable change estimates may allow clinicians to determine whether an improvement or decline in performance is greater than the expected improvement or decline due to measurement error in young adults.

## Introduction

Impaired balance and postural stability can occur in association with advanced age and a broad range of neurological disorders and neurodegenerative diseases, following traumatic brain injury, and as the result of acute or chronic orthopedic problems. The Balance Error Scoring System (BESS) is the most commonly used standardized clinical balance assessment following concussion among the general population (1, 2), in athletes (3–6), and in military service members (7). Some normative data for the test have been published across the lifespan (8–12). The BESS is also used in research relating to chronic ankle instability (11, 12) anterior cruciate ligament reconstruction (13), exercise-induced dehydration (14), exposure to toxins, such as diesel fuel exhaust (15), and in interventions to improve balance in youth athletes (16–18), adults who are obese (19), older adults (20–24), and older adults with Parkinson's disease (25). In a recent study, older adults who could not complete a simple 10-second one-leg balance stance had lower survival rates over a median of 7 years, even when controlling for age, sex, obesity, and clinical comorbidities, such as coronary artery disease, hypertension, and diabetes (26).

The BESS involves three stances, including a double leg stance (feet together), single leg stance (non-dominant leg), and tandem stance (dominant leg forward), performed on both a firm surface and a foam surface (27). Examinees stand with their hands on their hips and eyes closed for 20 s while the examiner counts the number of “errors,” up to a maximum of 10 errors. Errors including lifting hands off hips, opening eyes, stepping, stumbling, or falling, remaining out of the test position for 5 s, moving hip into more than 30 degrees of flexion or abduction, or lifting forefoot or heel. A score of 10 is also given to a person who cannot maintain the proper stance for at least 5 s throughout the 20 s period. There is subjectivity in the scoring of errors on the BESS, and the BESS has moderate test-retest reliability (intraclass correlation [ICC] = 0.57–0.74) (28).

The use of digital technology in the assessment of postural stability may improve measurement precision and accuracy and may reduce variability associated with differences in how examiners count errors on the BESS (i.e., subjectivity). Thus, numerous studies have investigated ways to instrument the BESS test with wearable accelerometers (29, 30), pressure platforms (31, 32), or force plate systems (33, 34). However, many of these devices are not widely accessible. The Sway Balance Mobile Application (Sway Medical, Inc., Tulsa OK, USA) (35–41), on the other hand, assesses postural stability using the tri-axial accelerometer built into mobile devices. The examinee performs a suite of five balance stances while holding the mobile device at mid-sternum. A proprietary algorithm detects and interprets motion using the accelerometers built into the mobile device and generates a balance score (36). This suite of five balance stances includes conditions similar to the BESS test, although single leg and tandem stances are performed with both dominant and non-dominant legs and all stances are performed with eyes closed and on a firm surface only (36, 37). Prior studies have administered in-person assessments of the Sway Balance Mobile Application and supported validity compared to the standardized clinical balance assessment tool BESS (*r* = 0.77) (36) and the BIODEX Balance System SD (*r* = 0.63 and *r* = −0.70) (35, 38). Amick and colleagues reported adequate test-retest reliability of the Sway Balance Mobile Application in a study that administered three separate in-person testing sessions spaced at least one-week apart (F (5, 115) = 0.673, *p* = 0.65, ICC (3,1) = 0.76, SEM = 5.39). Similar to the current study, each testing session included two trials (week 1 also included a familiarization trial) of the Sway Balance assessment and each session was at least 7-days apart (37).

Mobile devices are highly prevalent in industrialized nations today, so the Sway Balance Mobile Application may address accessibility concerns associated with other types of devices used for balance testing – allowing for widespread implementation, including remote monitoring by a clinician from a patient's home. There is growing interest in devices/applications used for telehealth as the COVID-19 pandemic highlighted the importance of telehealth to deliver care to patients (42). However, psychometric support is necessary prior to widespread implementation of remote balance tests. As noted above, prior work has examined test-retest reliability of the Sway Balance Mobile Application with in-person assessments (36, 37), but not when administered remotely from people's homes using their own devices. Thus, the purpose of this study was to examine test–retest reliability and reliable change estimates for Sway Balance tests administered remotely from the participant's home in a sample of community-dwelling adults. We hypothesized that test–retest reliability estimates would indicate good test-retest reliability (ICC ≥ 0.75). We also explored the association between age and sex with Sway Balance scores.

## Materials and methods

### Participants

Participants were recruited and enrolled as part of a study examining the test-retest reliability of the Sway Balance and Cognitive tests. We report findings relevant to Sway Balance tests herein; outcomes from Sway Cognitive tests, including reaction time measures are reported elsewhere (43). There were 61 adults, aged 18 and older, who were recruited and enrolled in the study. Exclusion criteria included medical conditions potentially affecting balance (i.e., musculoskeletal injury, neurological dysfunction, uncorrected vision, or a vestibular condition). Participants were also excluded if they could not use the Sway Balance Mobile Application (i.e., if they did not have a mobile device capable of downloading the application) or if they could not maintain video conferences on a second device during the testing session for video monitoring. As noted in our prior work, of the 61 participants who enrolled in the study, one withdrew from the study due to unforeseen medical issues, one withdrew due a time commitment, and four were removed due to equipment failure that prevented their data from being recorded (43). All participants provided written informed consent and the study procedures were approved by the university's Institutional Review Board.

### Procedures

Because the study was conducted during the COVID-19 pandemic, Sway Balance tests were administered remotely, on participants' personal mobile devices (iOS version 9.3 or higher or Android version 7.0 or higher), while using video-based virtual connections on a second personal device. Participants completed Sway Balance tests during one session each week, for three consecutive weeks. A minimum of 7 days and no more than 10 days lapsed between each of the weekly sessions. Consistent with manufacturer recommendations, within each test session, participants underwent two trials of the Sway Balance protocol, including one additional familiarization (practice) trial in Week 1 (i.e., three total trials in Week 1). The means of the two trials for each weekly session were used for analyses, leading to three data points (i.e., Week 1, Week 2, and Week 3) for each stance and for the Sway Balance composite score.

The Sway Balance Mobile Application protocol consists of five stances, each performed for 10 s; all stances were performed barefoot on a firm surface with eyes closed. Participants were instructed to hold their mobile device with both hands against the center of their chest. Stances included a double leg stance (feet together), a single leg stance (right foot), a single leg stance (left foot), a tandem stance (right foot in front), and a tandem stance (left foot in front). Instructions for each stance were presented on the mobile device screen before the start of each stance. At the end of each stance, the device made a noise (if the device settings allowed) or vibrated to indicate the test was complete. At the end of all stances, a proprietary algorithm designed by the manufacturer provided a Sway Balance score, ranging from 0 to 100, for each stance, as well as an overall composite score, which was the average of the five individual stances. A higher score indicates better balance.

### Statistical analyses

We first tested for systematic error (e.g., practice effects) using a Friedman's ANOVAs and then calculated ICCs using a mean-rating, absolute-agreement, 2-way mixed effects model to examine test-retest reliability. ICCs can be interpreted as the proportion of variance in observed scores that can be ascribed to true score variance. In other words, if the ICC is. 75, then 75% of the observed score variance results from true score variance and 25% results from error. Based on the 95% confident interval of the ICC estimate, ICC < 0.5 indicate poor test-retest reliability, 0.5 < ICC < 0.75 indicate moderate test-retest reliability, 0.75 < ICC < 0.9 indicate good reliability, and ICC > 0.9 indicate excellent reliability (44).

To examine how closely the reliable change estimates align with the actual distribution of difference scores, we calculated the natural distribution of the difference scores for Week 2 minus Week 1. For example, a 10% difference score refers to the change in score from Week 1 to Week 2 that occurs in 10% of the full sample. Then, we calculated reliable change scores from Week 1 to Week 2 at 70%, 80%, and 90% confidence intervals (*z *= 1.04, 1.28, and 1.64, respectively). Finally, we compared Sway Balance scores by sex and age. Because the Sway Balance scores did not meet the normality assumptions, we used non-parametric tests. Specifically, we used Mann-Whitney tests to examine for sex differences in outcome measures and Spearman's Rho Correlations to determine the associations between age and the balance scores. For all tests, statistical significance was set *a priori* at *p *< 0.01 to adjust for multiple comparisons (i.e., analyses across multiple stances).

## Results

The final sample included 55 adults (mean age = 26.7 ± 9.9 years, interquartile range = 20–30, range = 18–58; 38 [69%] women; 49 [89%] used an iPhone, 1 [2%] used an Android device, 2 [4%] used a Motorola device, 3 [5%] used a Samsung device). Descriptive information for each stance and for the Sway Balance composite score is presented in Table 1. The Friedman's ANOVAs were all nonsignificant (Table 2), suggesting that Sway Balance scores did not differ across Week 1, Week 2, and Week 3. Test-retest reliability for the Sway Balance composite score across three weeks was. 88 (Table 3). Test-retest reliability for individual stances ranged from. 62 to. 83 (all *p*s < 0.001).

**Table 1:** Descriptive statistics for the Sway Balance scores.

Sway Stances	N	Mean	SD	Md	IQR	Range
**Week 1**						
Balance – Overall	55	84.91	12.46	88.73	76.67–94.67	47.48–99.02
Feet Together	55	95.43	9.51	98.72	95.06-99.76	37.44–100.00
Tandem Stance Right Foot Forward	55	89.03	15.08	94.58	83.46–97.94	23.78–99.80
Tandem Stance Left Foot Forward	55	89.37	11.95	94.13	83.84–97.52	45.67–99.68
Single Leg Right	55	73.86	24.26	82.96	58.5–93.43	0.00–98.84
Single Leg Left	55	76.85	24.06	86.28	64.03–94.51	0.00–99.00
**Week 2**						
Balance – Overall	55	86.53	10.76	89.85	81.91–93.15	55.00–99.45
Feet Together	55	94.46	8.81	97.79	94.43–99.71	61.90–100.00
Tandem Stance Right Foot Forward	55	88.30	17.50	94.59	88.29–98.01	4.81–99.99
Tandem Stance Left Foot Forward	55	92.01	8.87	95.11	86.56–98.4	58.38–99.90
Single Leg Right	55	78.79	20.02	86.33	68.98–92.89	15.03–99.69
Single Leg Left	55	79.11	18.19	84.45	66.85–92.74	22.97–99.30
**Week 3**						
Balance – Overall	55	85.93	11.23	88.12	76.86–94.27	47.44–99.01
Feet Together	55	93.58	10.40	98.24	93.15–99.72	55.89–100.00
Tandem Stance Right Foot Forward	55	89.42	11.92	93.92	86.42–98.84	50.94–99.97
Tandem Stance Left Foot Forward	55	91.23	11.23	94.22	91.16–97.67	44.87–99.97
Single Leg Right	55	76.41	22.34	83.33	68.11–93.21	0.00–99.93
Single Leg Left	55	78.98	20.73	87.72	68.63–94.16	12.18–99.95

IQR, interquartile range; Md, median; N, sample size; SD, standard deviation.

**Table 2:** Friedman's ANOVA results.

Sway Stances	*Χ* ^2^_F_	df	*p*
Balance – Overall	1.35	2	0.51
Feet Together	2.82	2	0.25
Tandem Stance Right Foot Forward	1.35	2	0.51
Tandem Stance Left Foot Forward	3.13	2	0.21
Single Leg Right	0.69	2	0.71
Single Leg Left	0.47	2	0.79

ANOVA, analysis of variance; df, degrees of freedom.

**Table 3:** Test-retest reliability.

Sway Stances	ICC	95% CI	F Test with True Value 0			
		[lower, upper]	Value	df1	df2	*p*
**ICC for Weeks 1-3**						
Balance – Overall	0.88	0.81, 0.92	8.13	54	108	<0.001
Feet Together	0.72	0.56, 0.83	3.58	54	108	<0.001
Tandem Stance Right Foot Forward	0.80	0.68, 0.88	4.89	54	108	<0.001
Tandem Stance Left Foot Forward	0.69	0.51, 0.81	3.23	54	108	<0.001
Single Leg Right	0.81	0.71, 0.88	5.34	54	108	<0.001
Single Leg Left	0.83	0.74, 0.90	5.90	54	108	<0.001
**ICC for Weeks 1-2**						
Balance – Overall	0.82	0.69, 0.90	5.55	54	54	<0.001
Feet Together	0.76	0.59, 0.86	4.15	54	54	<0.001
Tandem Stance Right Foot Forward	0.77	0.61, 0.87	4.31	54	54	<0.001
Tandem Stance Left Foot Forward	0.62	0.35, 0.78	2.67	54	54	<0.001
Single Leg Right	0.69	0.47, 0.82	3.26	54	54	<0.001
Single Leg Left	0.74	0.55, 0.85	3.82	54	54	<0.001

Results of test-retest ICC Calculation for weeks 1-3 using mean-rating, absolute-agreement, 2-way mixed effects model and for weeks 1-2 using mean-rating, absolute-agreement, 2-way mixed effects model. CI, confidence interval; df, degrees of freedom; ICC, intraclass correlation coefficient.

The natural distributions of difference scores (Week 2 – Week 1) are presented in Table 4, and reliable change estimates are presented in Table 5. At the 80% confidence interval, preliminary reliable changes estimates were 9 points (i.e., 8.95) points for the Sway Balance composite score (see Table 5). This means that improvement or worsening by that many points, or more, can be interpreted as unlikely to be the result of measurement error because only approximately 10% of people will show that degree of improvement and 10% will show that degree of worsening.

**Table 4:** Interpreting change based on the natural distribution of week 2 – week 1 difference scores.

	Worse/Lower Scores				Improved/Higher Scores		
Sway Stances	10%	15%	20%	Normal Variability	20%	15%	10%
Balance – Overall	−7.48	−5.44	−4.77	−4.77, 6.73	6.73	8.46	10.97
Feet Together	−9.19	−4.02	−2.95	−2.95, 1.88	1.88	2.63	4.98
Tandem Stance Right Foot Forward	−12.33	−5.41	−3.33	−3.33, 6.9	6.90	9.44	14.78
Tandem Stance Left Foot Forward	−7.29	−6.05	−4.76	−4.76, 7.75	7.75	10.44	16.82
Single Leg Right	−22.27	−13.28	−7.59	−7.59, 19.76	19.76	24.09	29.50
Single Leg Left	−17.36	−13.98	−8.48	−8.48, 10.02	10.02	12.72	24.65

The difference scores in this table were derived by subtracting the Week 1 scores from the Week 2 scores. The 10% score refers to the difference score that occurs in 10% or fewer of the total sample, and the 20% difference score refers to the difference score that occurs in 20% or fewer of the total sample. Normal variability represents the range of difference scores obtained by 60% of the sample. For example, a difference score of 10.97 on the Sway Balance - Overall represents improved performance that is seen in 10% or fewer of the sample. A difference score of −5.44 on the Sway Balance - Overall represents a worsening in performance that is seen in 15% or fewer of the sample. For all tests, higher scores denote better performance.

**Table 5:** Reliable change for Sway Balance scores based on weeks 1–2 ICCs.

Sway Stances	M (SD1)	M (SD2)	ICC	SEM1	SEM2	SE_diff_	0.70 CI	0.80 CI	0.90 CI
Balance – Overall	84.91 (12.46)	86.53 (10.76)	0.82	5.29	4.57	6.99	±7.27	±8.95	±11.46
Feet Together	95.43 (9.51)	94.46 (8.81)	0.76	4.66	4.32	6.35	±6.60	±8.13	±10.41
Tandem Stance Right Foot Forward	89.03 (15.08)	88.3 (17.5)	0.77	7.23	8.39	11.08	±11.52	±14.18	±18.17
Tandem Stance Left Foot Forward	89.37 (11.95)	92.01 (8.87)	0.62	7.37	5.47	9.18	±9.55	±11.75	±15.06
Single Leg Right	73.86 (24.26)	78.79 (20.02)	0.69	13.51	11.15	17.52	±18.22	±22.43	±28.73
Single Leg Left	76.85 (24.06)	79.11 (18.19)	0.74	12.27	9.28	15.38	±16.00	±19.69	±25.22

CI, confidence interval; ICC, intraclass correlation coefficient; M, mean; SD, standard deviation; SE_diff_, standard error of the difference; SEM, standard error of measurement.

In this relatively young, healthy population, women had higher mean Sway Balance scores across most conditions, although findings did not reach statistical significance (Table 6). Similarly, bivariate correlations showed no statistically significant associations between age and Sway Balance scores, although scores declined with age (Spearman's Rho = −0.05 to −0.34, all *p*s > 0.01, Table 7).

**Table 6:** Sex differences in Sway Balance scores for week 1.

Sway Stances	Women M (SD)	Men M (SD)	Mann-Whitney *U*	*p*
Balance – Overall	86.72 (11.30)	80.86 (14.27)	420	0.08
Feet Together	94.95 (10.82)	96.52 (5.7)	349	0.64
Tandem Stance Right Foot Forward	90.68 (13.41)	85.33 (18.17)	418	0.08
Tandem Stance Left Foot Forward	91.58 (9.33)	84.42 (15.58)	428	0.06
Single Leg Right	77.11 (25.34)	66.6 (20.5)	446	0.03
Single Leg Left	79.28 (21.44)	71.42 (29.06)	367	0.43

M, mean; SD, standard deviation.

**Table 7:** Spearman's Rho correlations between age and Sway Balance scores for week 1.

	Total Sample *N* = 55		Women *N* = 38		Men *N* = 17	
**Sway Stances**	r	*p*	r	*p*	r	*p*
Balance – Overall	−0.13	0.35	−0.03	0.87	−0.26	0.32
Feet Together	−0.06	0.66	0.06	0.73	−0.45	0.07
Tandem Stance Right Foot Forward	−0.34	0.01	−0.34	0.04	−0.27	0.29
Tandem Stance Left Foot Forward	−0.25	0.07	−0.15	0.38	−0.39	0.13
Single Leg Right	−0.05	0.73	0.06	0.74	−0.10	0.70
Single Leg Left	−07	0.63	0.05	0.75	−0.37	0.15

## Discussion

The purpose of this study was to examine test-retest reliability and provide reliable change estimates for Sway Balance tests administered remotely in a sample of relatively young, healthy community-dwelling adults. Our primary findings were that test-retest reliability was good across Weeks 1 through 3 (Overall Sway Balance composite score, ICC = 0.88; test-retest reliability was lower for individual stances, ICC = 0.62–0.83, Table 3). In addition, the preliminary reliable change estimates (Table 5) may allow researchers and clinicians to interpret change scores relative to expected measurement error. Static balance and postural stability are commonly assessed following concussion in athletes (3–6), and this testing has other potential applications such as measuring functional performance in association with chronic ankle instability (11, 12) or anterior cruciate ligament reconstruction (13). There might also be benefits in assessing static balance as part of one's routine health assessment (20–24), in evaluating risk of fall in older adults (24, 26), or in relation to neurodegenerative diseases such as Parkinson's disease (25). Research is needed to establish reliability and validity in older adults to inform testing in these populations.

The preliminary reliable change estimates based on the natural distribution of difference scores, illustrated in Table 4, and the reliable change formula, illustrated in Table 5, are similar but not identical. This is because the distributions of balance scores at test and retest are not precisely normal, and there is a small practice effect, on average. The natural distribution of difference scores can be used to examine how the computed reliable change estimates apply to the present sample. Using the 70% confidence interval, reliable change in Table 5 is defined as 8 points. Using that confidence interval for the natural distribution of different scores, reliable change would be defined as a worsening of 6 points and an improvement of 9 points, so the reliable change estimate is roughly consistent with the difference scores in the present sample. Similar results can be seen for the 80% confidence interval, where reliable change is defined as 9 points. There is no definitive rule for how to define reliable change, and the estimate chosen can be tailored to the specific research or clinical context. In a situation where it is a high priority to detect change if it has occurred (i.e., higher sensitivity to change), then a lower change value can be selected with the understanding that there will be a greater risk for false positives (lower specificity). Similarly, if it is a high priority to minimize the risk of false positives, then a higher change score cut off can be selected.

### Sway Balance Mobile Application testing

The Sway Balance Mobile Application testing was developed to assess postural stability following mTBI/concussion (41), and early work suggests that outcome measures from the Sway Balance application were similar to those obtained on a BioDex Balance System SD (Balance System™ SD, Shirley, NY, USA) (35, 38), and were correlated with clinician rated BESS scores (36, 38). However, instrumented balance tests that can be administered remotely, such as Sway Balance, have potential applications beyond concussion assessment. A pilot study of 24 young healthy adults suggested good test-retest reliability (ICC = 0.76) of Sway Balance tests performed in-person at least 7 days apart (37). More recently, normative data have been reported in pediatric and youth athletes 9–21 years old (39, 40). Among these youth athletes, age and sex were important factors and authors provided age- and sex-based normative values for Sway Balance scores (40). This study adds to the growing body of literature describing the strengths and limitations of the Sway Balance Mobile Application by providing test-retest reliability and preliminary reliable change estimates in a sample of primarily young, healthy community-dwelling adults over one to three weeks. Although more research is needed, early data suggest this application may be a clinically useful tool for evaluating postural stability over time – as indicated by better test-retest reliability than clinician rated BESS scores (28), even when administered remotely. It is well established that postural sway is greater in children and in older adults (>65 years old) (45–47), with age-related balance declines being more apparent in the 4^th^ or 5^th^ decade. However, the participants recruited herein were, on average, 26.7 ± 9.9 years old with very few participants above age 40 years (only 13% of the sample). Therefore, age-based normative data are likely important when examining Sway Balance composite scores across the lifespan. The influence of sex on balance is somewhat controversial, but sex differences are often absent during quiet standing activities (48–51), such as the stances included in the Sway Balance protocol. Nonetheless, sex differences have been observed in prior Sway Balance studies (39, 40), so more research is needed in larger samples to determine whether sex-based normative data are needed. Ultimately, this study, even when administered in a remote telehealth approach, supports prior work suggesting the benefit of instrumented balance assessments vs. clinician scoring for improving reliability. Further, a primary benefit of these instrumented assessments is that postural sway can be more accurately and consistently recorded compared to human raters detecting errors.

### Importance of remote assessments

The ability to remotely assess examinees in their own home is also an advantage. In this study, Sway Balance tests were administered on participants' personal mobile devices, using video-based virtual connections, and test-retest reliability was still moderate-to-good. Telehealth, including health services provided using audio/video technology, not only provides access to rural or underserved communities, but is also becoming more commonplace since the COVID-19 pandemic. In addition, where more frequent assessments are advantageous such as fall-risk intervention for older adults (24, 26) and person's with a neurodegenerative disease (i.e., Parkinson's disease) (25), or with mTBI/concussion management for tracking a patient's recovery (3–6), telehealth options may increase patient satisfaction, provide efficient and quality care, and also minimize healthcare costs.

### Limitations

The current study has several limitations. First, participants included primarily younger, community-dwelling adults, so findings may not be generalizable to other populations, including older adults. Relatedly, the age distribution was such that most participants were in their 20s or 30s, with only a few in their 50s (sample mean age = 26.7, SD = 9.9 years). Given the age IQR in our study (20–30 years), together with important age-related changes in balance, we recommend that results are generalized primarily to young rather than middle-aged or older adults. Second, participants were tested remotely using their own mobile device (requiring a smart phone with either an iOS version of 9.3 or higher or an Android version 7.0 or higher), so testing in other environments may differ. Third, the sample size was relatively small (*N* = 55), which limits the robustness of the results. Fourth, there are limitations with comparing findings to other studies using other testing batteries. Notably, the Sway Balance protocol involves single leg testing performed on each leg, as well as two different tandem stances (one with right foot in front, one with left foot in front), which are not the same as standard BESS or other clinical balance assessment protocols. Additionally, each stance duration was only 10 s. This differs from other clinical balance assessment protocols, which are typically 20–30 s, and is shorter than the time typically used for some more advanced analysis methods, such as complexity measures (52, 53). Fifth, the aim of the study was to examine test-retest reliability; future work should examine the validity and accuracy of these outcome measures for remote assessment. Finally, test-retest reliability was only assessed over three weeks; longer test-retest reliability may be more applicable in some clinical and research contexts.

## Conclusion

In summary, we report our preliminary results suggest good test–retest reliability of the Sway Balance composite score in a sample of primarily young, healthy community-dwelling adults. Reliable change estimates may allow clinicians to determine whether an improvement or decline in performance is greater than the expected improvement or decline due to measurement error in this population. For example, change scores of nine points or more were uncommon in the present study and might be a preliminary indicator of reliable change in young adults. Larger replication studies are needed to define reliable change more definitively on this balance test. In the meantime, the Sway Balance Mobile Application does appear to have good test-retest reliability, even when administered remotely, and may be useful for balance and postural stability assessments following traumatic brain injury or orthopedic problems in young adults.

## Data Availability

The original contributions presented in the study are included in the article/Supplementary Material, further inquiries can be directed to the corresponding author/s.

## Appendix A. Raw data for balance testing.

**Table:**

			Overall			Feet Together			Tandem - Right			Tandem - Left			Single Leg - Right			Single Leg - Left		
ID	Age	Gender	W1	W2	W3	W1	W 2	W 3	W 1	W2	W 3	W 1	W2	W3	W 1	W 2	W3	W1	W2	W3
1	20	M	97.64	97.84	98.83	98.55	97.64	99.06	95.82	98.97	99.64	98.37	98.92	99.28	97.33	96.02	98.42	98.14	97.67	97.79
2	22	M	92.83	87.39	85.61	98.39	93.63	84.08	97.30	90.49	89.75	96.75	91.97	77.93	77.22	68.98	82.70	94.51	91.87	93.58
3	22	M	86.46	94.90	76.39	99.59	97.60	97.67	90.38	98.18	73.02	92.92	98.66	73.61	65.06	88.75	68.55	84.36	91.32	69.12
4	28	M	89.36	89.11	87.37	99.32	97.51	99.20	81.41	87.90	77.71	94.56	92.08	97.48	82.78	84.46	83.91	88.72	83.61	78.58
5	24	M	90.79	90.53	95.56	98.76	85.79	97.02	97.54	97.88	98.84	96.10	96.65	98.63	74.48	88.02	93.37	87.08	84.35	89.97
6	34	M	68.45	59.08	74.58	96.97	99.25	92.74	75.01	54.41	72.26	71.06	93.81	86.02	58.50	24.60	59.90	40.71	23.37	62.00
7	50	M	91.61	90.72	91.30	95.48	95.69	98.58	85.53	92.94	87.71	93.37	86.23	92.04	90.88	94.45	90.31	92.82	84.30	87.86
8	31	M	87.43	91.50	98.39	93.21	79.73	94.05	86.02	88.29	99.93	89.23	96.38	99.97	75.45	97.14	99.93	93.27	95.99	98.07
9	23	M	61.65	87.99	73.97	76.37	93.81	96.93	83.19	98.66	87.39	59.43	89.11	91.16	44.88	77.43	50.55	44.37	80.93	43.82
10	23	M	76.67	89.41	76.86	97.57	98.34	99.18	91.61	93.47	96.16	83.52	90.56	86.34	75.83	78.08	48.56	34.81	86.59	54.08
11	43	M	48.25	55.00	47.44	91.62	67.15	72.15	66.43	78.49	50.94	62.67	58.38	66.42	20.54	48.00	22.56	0.00	22.97	25.16
12	19	M	60.66	55.23	69.69	98.18	96.82	98.24	23.78	41.57	59.10	83.84	59.38	75.75	42.78	15.03	52.81	54.73	63.37	62.56
13	19	M	94.44	89.85	94.18	99.74	99.77	95.99	95.28	68.58	96.84	89.04	96.95	92.33	94.16	93.83	89.90	94.00	90.13	95.85
14	49	M	68.18	77.97	87.77	99.60	99.99	99.74	97.39	98.63	98.12	45.67	80.69	96.69	55.03	52.95	75.49	43.21	57.57	68.85
15	20	M	86.46	93.15	73.05	99.99	99.80	99.25	99.05	98.80	98.37	99.58	99.58	99.00	45.76	84.80	0.00	87.94	82.75	68.63
16	25	M	84.66	90.34	77.74	97.63	99.62	97.43	90.58	89.04	79.03	85.25	95.71	90.95	69.67	89.44	81.01	80.20	77.91	40.27
17	23	M	89.08	91.46	92.26	99.87	99.41	99.51	94.41	92.73	96.47	93.86	94.06	91.78	61.97	86.33	85.85	95.30	84.76	87.71
18	20	W	91.81	88.94	94.58	100.00	99.74	99.83	98.92	91.89	96.15	95.42	99.15	93.99	95.24	89.67	94.78	69.47	64.29	88.17
19	21	W	94.73	89.61	93.46	98.87	97.26	96.37	98.83	95.64	93.92	97.62	92.97	96.70	83.18	83.32	85.78	95.15	78.87	94.54
20	22	W	89.61	92.25	94.03	96.85	93.78	95.76	94.58	91.72	95.88	89.50	98.40	96.02	83.80	92.89	91.88	83.34	84.45	90.63
21	19	W	90.21	87.67	81.97	98.72	99.71	99.72	99.28	96.40	86.42	79.88	81.69	50.06	87.17	82.88	84.16	86.01	77.69	89.49
22	21	W	95.00	99.45	98.85	99.84	99.86	97.68	98.96	99.62	99.97	99.59	99.72	99.90	87.77	98.74	98.45	88.87	99.30	98.26
23	58	W	73.90	83.19	80.60	95.06	99.86	99.84	78.29	93.09	72.58	81.54	97.27	97.92	55.34	66.35	76.56	59.28	59.39	56.13
24	24	W	93.30	94.65	78.45	99.96	98.76	93.96	92.52	95.15	69.73	94.13	95.11	73.79	87.15	92.27	65.51	92.77	91.98	89.27
25	21	W	90.30	77.15	89.21	97.47	96.33	99.92	94.58	88.33	93.71	96.56	86.56	97.29	82.96	59.06	79.00	79.93	55.47	76.15
26	19	W	88.14	92.17	75.38	96.25	99.09	55.89	99.32	95.16	96.30	98.16	99.90	97.58	61.11	88.08	50.28	85.89	78.60	76.87
27	18	W	77.14	62.95	66.09	83.93	79.61	59.47	72.78	4.81	75.72	82.34	78.66	44.87	85.91	63.74	57.42	60.75	87.93	92.98
28	19	W	47.48	89.77	61.20	98.49	96.96	95.80	82.94	97.70	81.83	55.98	97.31	90.12	0.00	83.86	15.02	0.00	73.03	23.25
29	20	W	76.12	84.59	84.49	37.44	61.90	83.61	95.65	99.76	93.95	96.49	94.22	95.72	79.79	82.56	69.05	71.25	84.50	80.13
30	19	W	98.88	98.22	99.01	98.83	95.25	98.24	99.25	98.48	99.32	98.99	99.20	98.96	98.84	99.69	99.00	98.52	98.47	99.52
31	28	W	88.07	88.07	76.40	99.99	99.98	92.74	91.91	71.63	57.56	77.61	96.08	94.22	94.91	97.22	71.13	75.95	75.44	66.35
32	55	W	97.52	97.53	97.45	98.68	99.03	88.63	97.94	99.29	99.89	97.52	99.00	99.09	97.88	95.68	99.70	95.60	94.65	99.95
33	21	W	76.14	73.11	73.22	92.11	94.43	93.15	90.60	93.58	91.09	96.02	86.13	91.18	51.13	28.70	31.50	50.83	62.74	59.19
34	30	W	98.12	90.73	91.53	99.99	99.93	99.74	99.11	98.71	99.75	99.05	92.94	91.08	94.68	80.66	92.21	97.77	81.41	74.88
35	34	W	65.56	65.18	86.22	82.30	96.07	98.69	28.07	53.11	81.06	78.91	80.49	92.05	73.56	51.97	71.89	64.96	44.25	87.42
36	31	W	79.15	74.34	90.95	90.08	94.57	96.25	71.51	88.13	88.94	79.38	89.80	92.09	68.52	42.56	83.33	86.28	56.64	94.16
37	31	W	88.63	81.02	80.79	90.75	84.09	66.74	71.80	78.80	88.95	90.54	83.03	92.51	92.31	87.16	92.41	97.78	72.05	63.37
38	32	W	88.73	90.11	87.86	82.04	81.60	89.92	96.94	99.99	89.69	97.37	99.87	92.61	83.11	75.14	73.88	84.17	93.95	93.20
39	25	W	94.67	94.74	93.54	99.29	96.79	80.45	95.51	96.52	99.24	97.81	93.98	97.98	90.52	90.21	93.62	90.25	96.23	96.44
40	27	W	96.32	90.32	94.03	99.52	68.81	84.04	99.60	98.26	99.30	99.47	99.30	99.48	93.43	92.49	91.34	89.58	92.74	95.99
41	42	W	95.08	86.46	89.05	100.00	99.98	99.79	94.91	50.07	87.26	89.98	95.84	91.40	93.09	92.59	83.17	97.45	93.83	83.62
42	18	W	92.90	91.27	93.27	99.76	99.73	99.79	98.73	97.51	97.34	96.21	89.07	97.64	83.20	82.14	83.87	86.63	87.89	87.72
43	19	W	76.27	78.21	75.34	91.75	89.28	70.77	91.66	94.59	93.51	90.85	85.99	91.83	48.96	66.25	65.16	58.16	54.94	55.45
44	22	W	69.83	87.00	88.12	92.94	99.09	94.01	79.19	82.98	88.33	85.38	96.18	94.60	0.00	71.43	74.99	91.63	85.32	88.67
45	23	W	82.09	96.64	96.98	98.96	99.42	99.97	98.06	99.17	99.54	97.06	99.48	97.67	93.68	93.61	93.21	22.70	91.52	94.53
46	49	W	80.18	79.81	85.67	99.09	99.82	99.16	83.46	86.60	88.39	96.22	98.28	91.52	58.10	67.71	68.11	64.03	46.64	81.19
47	22	W	82.45	81.91	86.40	99.55	99.13	99.07	93.83	90.47	97.31	75.48	82.18	86.47	66.49	70.92	69.63	76.89	66.85	79.54
48	22	W	96.20	95.81	98.80	92.53	97.79	98.50	95.26	98.01	99.92	97.56	99.20	99.90	98.57	86.41	98.24	97.10	97.66	97.43
49	19	W	98.26	95.91	96.64	97.58	98.98	99.33	99.80	97.06	99.27	99.68	98.39	95.71	97.79	92.28	97.34	96.45	92.84	91.56
50	29	W	71.09	75.78	66.34	99.98	99.90	99.98	80.14	90.67	74.16	94.21	85.05	94.20	24.20	43.91	51.19	56.92	59.36	12.18
51	23	W	94.80	94.68	94.04	99.72	96.38	99.91	95.06	94.96	87.44	98.14	97.42	97.92	90.81	93.14	89.86	90.29	91.53	95.10
52	25	W	96.20	91.91	95.97	99.91	82.53	100.00	97.35	95.23	96.78	92.14	91.94	96.70	96.87	95.60	94.14	94.75	94.27	92.25
53	25	W	93.14	92.91	94.27	99.82	99.02	99.92	96.41	97.98	99.35	91.71	85.76	96.44	96.54	90.48	90.21	81.23	91.30	85.45
54	20	W	88.30	95.65	96.11	100.00	100.00	99.99	93.74	89.57	98.71	95.92	96.55	96.36	56.68	96.49	94.03	95.19	95.62	91.44
55	20	W	99.02	98.26	98.60	99.98	99.25	99.72	99.41	96.84	98.85	99.66	99.47	99.04	97.06	97.64	97.97	99.00	98.12	97.45

M, men; W, women; W1, week 1, W2, week 2, and W3, week 3.
